# Correlation between systemic inflammation markers and insulin resistance in type 2 diabetes mellitus patients and its diagnostic value analysis

**DOI:** 10.3389/fmed.2025.1737989

**Published:** 2026-01-20

**Authors:** Yongcheng Jiang, Meiping Yan, Hang Li, Yiwei Zhu, Zhijing Liang, Xinjing Chen, Yiming Deng, Lei He

**Affiliations:** 1 The Second School of Clinical Medicine, Southern Medical University, Guangzhou, Guangdong, China; 2 Department of Endocrinology, Zhujiang Hospital, Southern Medical University, Guangzhou, Guangdong, China; 3 The First School of Clinical Medicine, Southern Medical University, Guangzhou, Guangdong, China

**Keywords:** diabetes, diabetes mellitus, inflammation markers, insulin resistance in type 2, type 2 diabetes mellitus

## Abstract

**Background:**

Insulin resistance (IR) is a central pathophysiological feature of type 2 diabetes mellitus (T2DM) and is closely associated with chronic low-grade inflammation. Simple systemic inflammatory markers derived from routine laboratory testing may reflect this inflammatory–metabolic state; however, their clinical relevance in relation to IR remains incompletely defined.

**Methods:**

In this retrospective cross-sectional study, 2,177 patients with T2DM and 327 age- and sex-matched healthy controls were included. Insulin resistance was assessed using the homeostasis model assessment index (HOMA-IR), and patients with T2DM were classified as insulin-resistant or non–insulin-resistant based on established criteria. Systemic inflammatory markers, including the neutrophil-to-lymphocyte ratio (NLR), high-sensitivity C-reactive protein (hs-CRP), and white blood cell count (WBC), were analysed. Associations with IR were examined using correlation analysis and multivariable logistic regression. Receiver operating characteristic (ROC) curve analysis was performed to evaluate discriminative performance.

**Results:**

Levels of NLR, hs-CRP, and WBC were significantly higher in patients with T2DM than in healthy controls and were further elevated in insulin-resistant patients. All three markers were positively correlated with HOMA-IR, with NLR showing the strongest association (*r* = 0.280, *p* < 0.001). In multivariable logistic regression analysis, NLR remained independently associated with insulin resistance after adjustment for body mass index, glycated haemoglobin, and triglyceride levels. ROC analysis demonstrated that NLR had the highest area under the curve (AUC = 0.670), indicating modest discriminative ability, with higher sensitivity but lower specificity compared with hs-CRP and WBC.

**Conclusion:**

Systemic inflammatory markers, particularly NLR, are significantly associated with insulin resistance in patients with T2DM. Although the discriminative performance of NLR was modest, its simplicity, low cost, and availability from routine complete blood counts support its potential role as a complementary marker associated with IR rather than a standalone diagnostic tool. Prospective studies are needed to clarify temporal relationships and validate these findings across diverse populations.

## 1 Introduction

Insulin resistance (IR) is a core metabolic abnormality defined by reduced responsiveness of insulin-sensitive tissues to circulating insulin and plays a central role in the development of several metabolic disorders, including type 2 diabetes mellitus (T2DM), atherosclerotic cardiovascular disease, metabolic syndrome, and non-alcoholic fatty liver disease. Importantly, IR typically develops before the onset of overt hyperglycaemia, which represents the hallmark clinical feature of T2DM. During this early stage, compensatory hyperinsulinaemia occurs in an effort to maintain normal glucose levels; however, persistent metabolic stress gradually impairs pancreatic *β*-cell function, ultimately facilitating progression to overt diabetes. Accumulating evidence indicates that IR represents a key early event in the pathophysiology of both prediabetes and T2DM and contributes substantially to impaired glucose tolerance, dyslipidaemia, and increased adiposity. In this context, chronic low-grade inflammation has been increasingly recognized as a biological process closely associated with IR and the progression of T2DM (1).

A growing body of experimental and epidemiological studies suggests that inflammatory processes interfere with insulin signalling pathways and metabolic homeostasis. Circulating levels of pro-inflammatory mediators, including interleukin-6, tumour necrosis factor-*α*, C-reactive protein, and white blood cell count, are consistently elevated in individuals with diabetes mellitus, supporting a close link between systemic inflammation and insulin resistance (2, 3). These findings reinforce the concept that IR extends beyond a purely metabolic disorder and is closely connected to immune activation and inflammatory responses.

In clinical research, several systemic inflammatory markers have been widely studied, particularly in oncology, where they have demonstrated value in disease stratification, treatment monitoring, and prognostic assessment. Composite indices such as the systemic immune-inflammation index, platelet-to-lymphocyte ratio, C-reactive protein, and neutrophil-to-lymphocyte ratio have been shown to correlate with tumour burden, immune status, and clinical outcomes, especially in pancreatic cancer (4). These markers reflect the balance between inflammatory activity and immune regulation. Despite their broad application in oncological settings, their relevance in metabolic disorders, particularly IR and its related complications, has received comparatively limited investigation.

Among these indices, the neutrophil-to-lymphocyte ratio has attracted increasing attention as a simple and readily available marker of systemic inflammation derived from routine complete blood count testing (5). NLR reflects the balance between innate immune activity, represented by neutrophils, and adaptive immune regulation, represented by lymphocytes, thereby providing an integrated measure of inflammatory status. Elevated NLR levels have been reported in association with metabolic syndrome, cardiovascular disease, and T2DM, suggesting that this marker may capture chronic inflammatory states relevant to metabolic dysfunction (6, 7).

High-sensitivity C-reactive protein is another widely used indicator of systemic inflammation and is valued for its ability to detect low-grade inflammatory activity. Its clinical utility has been established in cardiovascular disease and other inflammatory conditions (8). However, the relative contribution of hs-CRP compared with cellular inflammatory indices such as NLR and white blood cell count in the context of insulin resistance remains incompletely understood, with prior studies reporting heterogeneous findings across different populations and study designs.

Although established methods for assessing insulin resistance, including clamp techniques and insulin-based indices, offer high specificity, they are labor-intensive and not easily implemented in routine clinical practice or large-scale screening. Therefore, there is ongoing interest in identifying simple and cost-effective markers that may be associated with IR. The present study was designed to compare selected systemic inflammatory markers among patients with T2DM with and without insulin resistance and healthy controls. By examining the associations between inflammatory indices and IR and evaluating their discriminative performance, this study aims to clarify the potential clinical relevance of routinely available inflammatory markers as correlates of insulin resistance, while acknowledging the limitations inherent to a cross-sectional study design.

## 2 Subjects and methods

### 2.1 Study design and study population

This retrospective observational study was conducted at Zhujiang Hospital of Southern Medical University. Adult patients diagnosed with type 2 diabetes mellitus (T2DM) according to the 1999 World Health Organization diagnostic criteria who attended the hospital between February 2014 and December 2024 were eligible for screening. During this period, a total of 2,177 patients with T2DM were initially identified from the hospital electronic medical record system.

To minimize potential confounding effects on systemic inflammatory markers, individuals were excluded if they had acute or critical illnesses, a documented history of cardiovascular disease, malignant tumours, Hodgkin’s lymphoma, acute or chronic leukaemia, or if they were receiving medications known to substantially affect inflammatory status, with the exception of standard oral hypoglycaemic agents or insulin therapy. After applying these exclusion criteria, 523 individuals were excluded from the initial T2DM cohort. The remaining patients constituted the final diabetic study population and were included in the analysis.

In parallel, a group of 327 apparently healthy individuals without diabetes was recruited as a reference control group. Healthy controls were frequency matched to the T2DM cohort by age and sex to reduce demographic imbalance between groups. Control participants were selected from individuals undergoing routine health examinations at the same institution during the study period and had no documented history of diabetes or major inflammatory or malignant diseases.

Demographic and clinical information, including age, sex, smoking status, comorbid conditions, hyperlipidaemia, family history of metabolic disease, and medication use, was extracted from electronic medical records. A flow diagram summarizing participant screening, exclusions, and final group allocation is provided to clarify the study population selection process.

### 2.2 Ethical considerations

The study protocol was developed in accordance with the principles of the Declaration of Helsinki and was approved by the Medical Ethics Committee of Zhujiang Hospital, Southern Medical University (Approval No. 2023HXKT14). Given the retrospective nature of the study and the use of anonymized clinical data, the requirement for individual informed consent was waived by the ethics committee.

### 2.3 Clinical and laboratory measurements

All clinical and laboratory assessments were performed after an overnight fast of at least 12 h. Blood pressure measurements were obtained in the early morning as part of routine clinical assessment using standard hospital procedures. Blood pressure variables were included for descriptive baseline characterization only and were not incorporated into inferential or regression analyses.

Fasting venous blood samples were collected for measurement of complete blood counts, lipid profiles including total cholesterol, high-density lipoprotein cholesterol, low-density lipoprotein cholesterol, and triglycerides, as well as high-sensitivity C-reactive protein, glycated haemoglobin, fasting plasma glucose, and fasting insulin. Fasting insulin concentrations were consistently expressed in mIU/L.

The neutrophil-to-lymphocyte ratio was calculated by dividing the absolute neutrophil count by the absolute lymphocyte count. White blood cell count was recorded as an additional indicator of systemic inflammation. These inflammatory markers were analysed in relation to insulin resistance in accordance with prior studies examining associations between inflammation and metabolic dysfunction.

### 2.4 Definition of insulin resistance

Insulin resistance was estimated using the homeostasis model assessment index (HOMA-IR), calculated as fasting insulin (mIU/L) multiplied by fasting plasma glucose (mmol/L) and divided by 22.5. Based on the “Expert Consensus on Clinical Issues Related to Insulin Resistance (2022 Edition)” issued by Chinese health authorities, patients with T2DM were classified into an insulin-resistant group (HOMA-IR ≥ 2.80) and a non–insulin-resistant group (HOMA-IR < 2.80). Healthy individuals without diabetes served as the control group.

### 2.5 Statistical analysis

Statistical analyses were conducted using SPSS software version 26.0. Continuous variables with a normal distribution were expressed as mean ± standard deviation and compared between groups using independent samples *t*-tests or analysis of variance, as appropriate. Variables with a skewed distribution were presented as medians with interquartile ranges and compared using the Mann–Whitney *U* test. Categorical variables were analysed using chi-square tests.

Given the non-normal distribution of HOMA-IR, Spearman’s rank correlation analysis was used to assess associations between inflammatory markers and insulin resistance. Receiver operating characteristic curve analysis was performed to evaluate the discriminative performance of inflammatory indices in distinguishing insulin-resistant from non–insulin-resistant patients. Binary logistic regression analysis was applied to examine adjusted associations between selected clinical and inflammatory variables and insulin resistance. Covariates were selected based on biological plausibility and evidence from prior literature. All statistical tests were two-tailed, and a *p* value < 0.05 was considered statistically significant.

## 3 Results

### 3.1 Baseline characteristics of the study population

Baseline demographic and clinical characteristics of the study population stratified into insulin-resistant, non–insulin-resistant, and healthy control groups are presented in Table 1. The three groups were comparable with respect to age, sex distribution, and smoking status, indicating an overall balanced baseline profile. Blood pressure measurements did not differ significantly among groups. Notably, variability in systolic and diastolic blood pressure was higher in the non–insulin-resistant group, which likely reflects heterogeneity inherent to retrospective clinical records, including differences in measurement timing and clinical context. As blood pressure variables were included for descriptive purposes only and were not incorporated into inferential or regression analyses, this variability does not affect the primary findings of the study.

**Table 1:** Baseline demographic and clinical characteristics of the study population.

Variable	Non–insulin-resistant group (*n* = 1,008)	Insulin-resistant group (*n* = 646)	Healthy controls (*n* = 327)	*p*-value
Age (years)	52.72 ± 14.48	51.59 ± 16.50	51.18 ± 18.27	0.142
Male sex, *n* (%)	549 (54.5)	372 (57.6)	178 (54.5)	0.192
Smoking history, *n* (%)	158 (14.2)	65 (15.1)	35 (13.2)	0.958
Body mass index (kg/m^2^)	22.88 ± 3.77	25.17 ± 7.86	22.98 ± 3.96	< 0.001
Systolic blood pressure (mmHg)	127.46 ± 36.95	128.58 ± 17.58	126.57 ± 17.00	0.579
Diastolic blood pressure (mmHg)	78.92 ± 41.83	79.77 ± 28.61	76.31 ± 10.42	0.407

Data are presented as mean ± standard deviation or number (percentage), as appropriate. Blood pressure measurements were obtained from routine clinical records and are reported for descriptive baseline characterization only. Variability in blood pressure values reflects heterogeneity inherent to retrospective clinical documentation, including differences in measurement timing and clinical context. Blood pressure variables were not included in inferential or regression analyses and therefore do not affect the primary conclusions of the study.

In contrast, body mass index differed markedly across groups. Individuals with insulin resistance exhibited significantly higher BMI values than both non–insulin-resistant patients and healthy controls, highlighting the close association between excess adiposity and insulin resistance.

### 3.2 Comparison of metabolic and laboratory parameters between patients with T2DM and healthy controls

Comparative analyses of metabolic and laboratory parameters between patients with type 2 diabetes mellitus and healthy controls are summarized in Table 2. As expected, patients with T2DM demonstrated substantially impaired glycaemic control, as reflected by higher fasting plasma glucose, glycated haemoglobin, fasting insulin, and HOMA-IR values. With respect to lipid metabolism, triglyceride levels were significantly elevated in the diabetic group, whereas total cholesterol, high-density lipoprotein cholesterol, and low-density lipoprotein cholesterol showed no significant differences, suggesting selective dysregulation of lipid components rather than a uniform alteration across the lipid profile.

**Table 2:** Comparison of metabolic and inflammatory parameters between patients with type 2 diabetes mellitus and healthy controls.

Variable	T2DM group (*n* = 1,654)	Healthy controls (*n* = 327)	*p*-value^*^
Triglycerides (mmol/L)	1.41 (1.24)	1.26 (0.95)	0.002
Total cholesterol (mmol/L)	5.10 ± 1.35	5.08 ± 1.43	0.736
HDL-C (mmol/L)	1.27 ± 0.42	1.29 ± 0.36	0.383
LDL-C (mmol/L)	3.13 ± 1.13	3.05 ± 1.17	0.340
Fasting insulin (mIU/L)	5.74 (4.57)	5.99 (4.41)	<0.001
Fasting plasma glucose (mmol/L)	8.62 ± 4.56	5.04 ± 1.16	<0.001
HbA1c (%)	7.79 ± 2.29	5.80 ± 0.71	<0.001
hs-CRP (mg/L)	1.78 (2.86)	1.36 (2.57)	<0.001
HOMA-IR	2.27 (1.56)	1.34 (1.02)	<0.001
Neutrophil-to-lymphocyte ratio	2.20 ± 1.78	1.74 ± 0.68	<0.001
Neutrophil count (×10^9^/L)	4.39 ± 2.55	3.65 ± 1.32	<0.001
Lymphocyte count (×10^9^/L)	2.22 ± 0.75	2.22 ± 0.71	0.913
White blood cell count (×10^9^/L)	7.29 ± 2.90	6.55 ± 1.84	<0.001

Data are presented as mean ± standard deviation for normally distributed variables and median (interquartile range) for non-normally distributed variables. ^*^p values were calculated using independent samples *t*-tests or Mann–Whitney *U* tests, as appropriate. HDL-C, high-density lipoprotein cholesterol; LDL-C, low-density lipoprotein cholesterol; HbA1c, glycated haemoglobin; hs-CRP, high-sensitivity C-reactive protein; HOMA-IR, homeostasis model assessment of insulin resistance.

Markers of systemic inflammation were consistently higher in patients with T2DM. Elevated hs-CRP, white blood cell count, and neutrophil-to-lymphocyte ratio collectively indicate an enhanced inflammatory milieu in diabetes compared with metabolically healthy individuals. In contrast, absolute lymphocyte counts were comparable between groups, suggesting that the observed increase in NLR was driven primarily by neutrophil predominance rather than lymphocyte depletion.

### 3.3 Metabolic and inflammatory profiles according to insulin resistance status in T2DM

Stratification of patients with T2DM according to insulin resistance status revealed pronounced differences in metabolic and inflammatory characteristics (Table 3). Insulin-resistant patients exhibited a distinctly more adverse metabolic profile, characterized by higher triglycerides, total cholesterol, low-density lipoprotein cholesterol, fasting insulin, fasting plasma glucose, glycated haemoglobin, and HOMA-IR values. Conversely, high-density lipoprotein cholesterol levels were significantly lower in this group, consistent with dyslipidaemia associated with insulin resistance.

**Table 3:** Metabolic and inflammatory profiles according to insulin resistance status in patients with type 2 diabetes mellitus.

Variable	Non-insulin-resistant group (*n* = 1,008)	Insulin-resistant group (*n* = 646)	*p*-value
Triglycerides (mmol/L)	1.26 (1.03)	1.75 (1.47)	<0.001
Total cholesterol (mmol/L)	5.01 ± 1.17	5.26 ± 1.58	<0.001
HDL-C (mmol/L)	1.31 ± 0.38	1.22 ± 0.46	<0.001
LDL-C (mmol/L)	3.07 ± 0.95	3.23 ± 1.37	0.007
Fasting insulin (mIU/L)	5.17 (3.82)	11.93 (7.82)	<0.001
Fasting plasma glucose (mmol/L)	7.23 ± 3.59	10.78 ± 5.04	<0.001
HbA1c (%)	7.35 ± 2.06	8.79 ± 2.48	<0.001
hs-CRP (mg/L)	1.67 (2.71)	3.36 (6.52)	<0.001
HOMA-IR	1.55 (1.06)	4.63 (3.27)	<0.001
Neutrophil-to-lymphocyte ratio	1.84 ± 0.77	2.77 ± 2.58	<0.001
Neutrophil count (×10^9^/L)	3.88 ± 1.43	5.18 ± 3.52	<0.001
Lymphocyte count (×10^9^/L)	2.26 ± 0.74	2.15 ± 0.77	0.004
White blood cell count (×10^9^/L)	6.82 ± 1.93	8.04 ± 3.84	<0.001

Data are presented as mean ± standard deviation for normally distributed variables and median (interquartile range) for non-normally distributed variables. HDL-C, high-density lipoprotein cholesterol; LDL-C, low-density lipoprotein cholesterol; HbA1c, glycated haemoglobin; hs-CRP, high-sensitivity C-reactive protein; HOMA-IR, homeostasis model assessment of insulin resistance.

Inflammatory markers further distinguished insulin-resistant from non–insulin-resistant patients. Levels of hs-CRP, white blood cell count, and NLR were all significantly elevated in the insulin-resistant group. These changes were accompanied by higher neutrophil counts and modestly reduced lymphocyte counts, reflecting a shift toward a pro-inflammatory cellular profile associated with impaired insulin sensitivity.

### 3.4 Association between inflammatory markers and insulin resistance

Spearman’s rank correlation analysis demonstrated significant positive associations between insulin resistance, as quantified by HOMA-IR, and all examined inflammatory markers. Among these, NLR showed the strongest correlation, followed by white blood cell count and hs-CRP. These findings suggest that systemic inflammatory activation is closely linked to the degree of insulin resistance, with cellular inflammatory indices exhibiting particularly robust associations.

### 3.5 Multivariable analysis of factors associated with insulin resistance

In multivariable logistic regression analysis adjusting for demographic and metabolic covariates, NLR remained significantly associated with insulin resistance alongside body mass index, glycated haemoglobin, and triglyceride levels. The results of the multivariable logistic regression analysis examining factors associated with insulin resistance are summarized in Table 4. In contrast, the associations for hs-CRP and white blood cell count were attenuated after adjustment, indicating that NLR may capture aspects of inflammation related to insulin resistance that are not fully reflected by these markers.

**Table 4:** Multivariable logistic regression analysis of factors associated with insulin resistance.

Variable	*p*-value	Odds ratio (OR)	95% confidence interval
Neutrophil-to-lymphocyte ratio	0.015	1.758	1.118–2.764
Triglycerides (mmol/L)	0.036	1.415	1.022–1.958
Body mass index (kg/m^2^)	<0.001	1.283	1.133–1.453
HbA1c (%)	<0.001	1.404	1.170–1.686

Multivariable logistic regression analysis examining factors associated with insulin resistance in patients with type 2 diabetes mellitus. Variables were selected based on univariate screening, biological plausibility, and prior evidence. Odds ratios represent adjusted associations derived from a cross-sectional model and do not imply causality.

### 3.6 Discriminative performance of inflammatory markers

**Figure 1:**
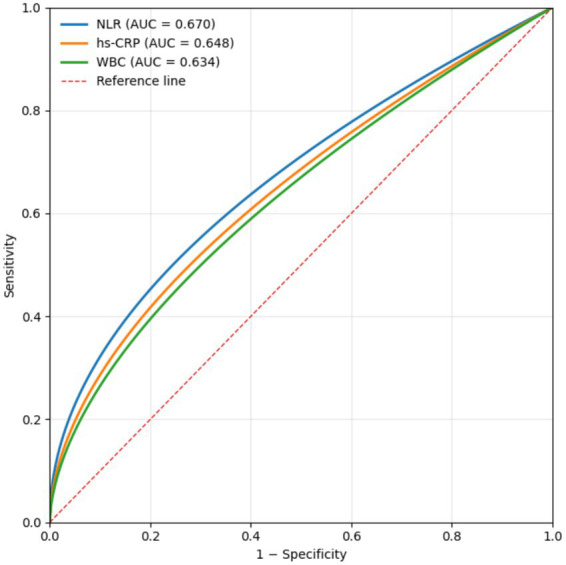
Receiver operating characteristic curves of systemic inflammatory markers for insulin resistance. Receiver operating characteristic (ROC) curves illustrating the discriminative performance of the neutrophil-to-lymphocyte ratio (NLR), high-sensitivity C-reactive protein (hs-CRP), and white blood cell count (WBC) in distinguishing insulin-resistant from non–insulin-resistant patients with type 2 diabetes mellitus. The areas under the curve indicate modest discriminative ability for all three markers. Among them, NLR exhibited a relatively higher sensitivity, whereas WBC demonstrated higher specificity. These findings suggest that systemic inflammatory markers are associated with insulin resistance but have limited standalone diagnostic utility.

Receiver operating characteristic curve analysis was used to assess the ability of inflammatory markers to discriminate between insulin-resistant and non–insulin-resistant patients with T2DM. NLR demonstrated the highest area under the curve, with relatively higher sensitivity but lower specificity compared with hs-CRP and white blood cell count. However, the magnitude of the AUC indicated only modest discriminative performance overall. These results suggest that while NLR may provide useful supplementary information, it should not be considered a standalone diagnostic marker for insulin resistance. Detailed ROC parameters are provided in Table 5 and Figure 1.

**Table 5:** Receiver operating characteristic (ROC) curve parameters of systemic inflammatory markers.

Marker	Area under the curve (AUC)	Cut-off value	*P* value	95% confidence interval	Sensitivity (%)	Specificity (%)
Neutrophil-to-lymphocyte ratio	0.670	1.73	<0.001	0.632–0.708	74.0	51.7
hs-CRP (mg/L)	0.648	3.28	<0.001	0.609–0.687	67.6	54.4
White blood cell count (×10^9^/L)	0.634	6.64	<0.001	0.594–0.675	51.4	72.7

Receiver operating characteristic curve analysis was performed to evaluate the ability of inflammatory markers to distinguish insulin-resistant from non–insulin-resistant patients with type 2 diabetes mellitus. Area under the curve values indicate modest discriminative performance and should be interpreted as associative rather than diagnostic.

## 4 Discussion

In this cross-sectional study, systemic inflammatory markers, including the neutrophil-to-lymphocyte ratio (NLR), high-sensitivity C-reactive protein (hs-CRP), and white blood cell count, were significantly higher in individuals with type 2 diabetes mellitus, particularly among those with insulin resistance, compared with non–insulin-resistant patients and healthy controls. A progressive increase in these markers from healthy participants to non–insulin-resistant and insulin-resistant groups was observed, suggesting a close association between systemic inflammation and worsening metabolic status. Consistent with this pattern, all three inflammatory indices were positively correlated with HOMA-IR, with NLR showing the strongest association, indicating a closer relationship with the degree of insulin resistance (1, 9, 10).

These findings align with extensive evidence linking chronic low-grade inflammation to insulin resistance and type 2 diabetes mellitus (1, 9). Although the precise biological mechanisms underlying insulin resistance are not fully elucidated, experimental and epidemiological studies consistently demonstrate that inflammatory activation accompanies metabolic dysfunction. Within the context of the present cross-sectional design, the observed relationships should be interpreted as associations rather than causal effects. The elevated inflammatory markers observed in insulin-resistant individuals likely reflect a metabolic–inflammatory state in which immune activation and impaired insulin sensitivity coexist, rather than a unidirectional causal pathway.

Alterations in leukocyte subpopulations observed in this study provide further insight into the inflammatory profile associated with insulin resistance. Insulin-resistant patients exhibited higher neutrophil counts alongside modest reductions in lymphocyte counts, resulting in an elevated NLR. This pattern is consistent with prior research in metabolic and inflammatory conditions, where chronic low-grade inflammation has been shown to influence both innate and adaptive immune responses (10). In this context, NLR represents an integrated index reflecting the balance between these immune pathways. The stronger correlation between NLR and HOMA-IR compared with hs-CRP and total white blood cell count suggests that cellular inflammatory responses may be more closely aligned with insulin resistance than isolated circulating protein markers.

Among the inflammatory indices examined, NLR demonstrated the strongest association with insulin resistance and the highest sensitivity in receiver operating characteristic analysis. However, its overall discriminative performance was modest, as indicated by an AUC of 0.67. This finding does not support the use of NLR as a standalone diagnostic marker for insulin resistance. Rather, NLR should be viewed as a readily available inflammatory indicator that may complement established metabolic assessments. Similar associations between elevated NLR and adverse metabolic or cardiovascular profiles have been reported in studies of coronary artery disease, malignancies, and diabetic complications, supporting its relevance as a general marker of systemic inflammatory burden (4, 6, 11).

The biological plausibility of the association between elevated NLR and insulin resistance is supported by experimental evidence linking hyperglycaemia and dyslipidaemia to immune activation. Metabolic disturbances characteristic of insulin resistance have been shown to promote neutrophil activation, adhesion, and migration, as well as the release of reactive oxygen species and inflammatory mediators that may further impair insulin signalling pathways (12, 13). At the same time, chronic inflammatory stress and metabolic dysregulation may contribute to relative lymphopenia through altered lymphocyte trafficking, apoptosis, or impaired cytokine signalling (14, 15). The resulting imbalance, reflected by an elevated NLR, captures a systemic inflammatory environment biased toward innate immune activation, which is increasingly recognized as detrimental to metabolic homeostasis (11, 16).

In multivariable analyses, NLR remained independently associated with insulin resistance after adjustment for established metabolic covariates, including body mass index, glycated haemoglobin, and triglyceride levels. While this finding underscores the robustness of the association, it should not be interpreted as evidence of causality. Instead, it suggests that NLR provides information related to insulin resistance that is not fully explained by traditional metabolic parameters alone. The concurrent associations observed for BMI, HbA1c, and triglycerides are consistent with a large body of literature demonstrating the close interplay between adiposity, dyslipidaemia, chronic hyperglycaemia, and insulin resistance (17, 18). Adipose tissue–derived cytokines and lipid-induced inflammatory pathways may contribute to a shared inflammatory–metabolic phenotype underlying these associations.

Receiver operating characteristic analysis further indicated that NLR exhibited higher sensitivity than hs-CRP and white blood cell count, although at the expense of lower specificity. This pattern suggests that NLR may be more effective in identifying individuals with insulin resistance but may also generate false-positive classifications. Consequently, its potential clinical value lies in its use as a supplementary or screening marker rather than a definitive diagnostic test. Given that NLR is derived from routine complete blood counts, its accessibility and low cost may make it useful for exploratory assessment in large populations or resource-limited settings when interpreted alongside other metabolic indicators (11, 16).

The relatively stronger performance of NLR compared with hs-CRP observed in this study may reflect differences in the biological processes captured by these markers. While hs-CRP primarily reflects hepatic acute-phase responses driven by interleukin-6, NLR captures dynamic changes in circulating immune cell populations, which may be more sensitive to the chronic, low-grade inflammatory environment characteristic of insulin resistance. Moreover, hs-CRP levels can be influenced by non-metabolic factors, including liver function and concurrent inflammatory conditions, potentially contributing to variability (8, 19).

Overall, the present findings demonstrate a significant association between systemic inflammatory markers, particularly NLR, and insulin resistance in individuals with type 2 diabetes mellitus. These results support the concept that inflammation and metabolic dysfunction are closely interconnected and highlight the potential value of incorporating simple inflammatory indices into the broader assessment of metabolic health. However, careful interpretation is required, and further prospective and longitudinal studies are necessary to clarify temporal relationships, explore underlying mechanisms, and determine whether changes in inflammatory markers over time parallel the development or improvement of insulin resistance in response to therapeutic interventions.

## 5 Conclusion

Several limitations of the present study should be considered when interpreting the findings. First, the retrospective and cross-sectional design precludes any inference regarding causal or temporal relationships between systemic inflammation and insulin resistance. The observed associations reflect coexistence rather than directionality, and it cannot be determined whether inflammatory activation precedes the development of insulin resistance or arises as a consequence of metabolic dysfunction. Although multivariable analyses were applied to adjust for major metabolic confounders, residual confounding related to unmeasured factors—such as physical activity, dietary patterns, psychosocial stress, or socioeconomic status—cannot be excluded.

Second, this study was conducted at a single tertiary center in China, which may limit the generalizability of the findings to other ethnic groups or populations with different genetic backgrounds, lifestyles, or healthcare settings. Ethnic- and region-specific differences in inflammatory responses and metabolic profiles may influence the observed associations, underscoring the need for validation in diverse geographic and demographic populations.

Third, the analysis focused on readily available inflammatory markers derived from routine laboratory testing. Other inflammatory mediators and cytokines, including interleukin-6, tumour necrosis factor-*α*, adiponectin, as well as emerging composite indices such as the hs-CRP/albumin ratio and monocyte-to-lymphocyte ratio, were not assessed. Inclusion of these markers in future prospective studies may provide deeper mechanistic insight into the inflammatory–metabolic interface and allow refinement of models examining the relationship between inflammation and insulin resistance.

Despite these limitations, this large-scale study demonstrates that systemic inflammatory markers, particularly the neutrophil-to-lymphocyte ratio, are significantly elevated and positively associated with insulin resistance in individuals with type 2 diabetes mellitus. Compared with hs-CRP and white blood cell count, NLR showed a stronger association with insulin resistance and higher sensitivity, although its discriminative performance remained modest, indicating limited standalone diagnostic utility. Given its simplicity, low cost, and availability from routine complete blood count testing, NLR may be considered a complementary marker associated with insulin resistance rather than a definitive diagnostic tool.

Future research should prioritize prospective and longitudinal designs to clarify temporal relationships between inflammation and insulin resistance and to determine whether changes in inflammatory markers parallel the progression or improvement of metabolic dysfunction. Multicenter studies involving diverse populations are also warranted to enhance external validity. In addition, investigating the dynamic behavior of NLR and other inflammatory indices in response to anti-diabetic therapies, particularly agents with known anti-inflammatory effects such as sodium–glucose cotransporter 2 inhibitors and glucagon-like peptide-1 receptor agonists, may further elucidate their potential clinical relevance (20, 21).

## Data Availability

The original contributions presented in the study are included in the article/supplementary material, further inquiries can be directed to the corresponding author/s.
